# The carbonic anhydrase of *Clostridium autoethanogenum* represents a new subclass of β-carbonic anhydrases

**DOI:** 10.1007/s00253-019-10015-w

**Published:** 2019-07-25

**Authors:** Bart Pander, Gemma Harris, David J. Scott, Klaus Winzer, Michael Köpke, Sean D. Simpson, Nigel P. Minton, Anne M. Henstra

**Affiliations:** 1 Clostridia Research Group, BBSRC/EPSRC Synthetic Biology Research Centre, School of Life Sciences, University of Nottingham, Nottingham, NG7 2RD UK; 2 Research Complex at Harwell, Rutherford Appleton Laboratory, Harwell Science and Innovation Campus, Didcot, OX11 0FA UK; 3 ISIS Spallation Neutron and Muon Source, Rutherford Appleton Laboratory, Harwell Science and Innovation Campus, Didcot, OX11 0QX UK; 4 School of Biosciences, University of Nottingham, Sutton Bonington Campus, Sutton Bonington, LE12 5RD UK; 5 LanzaTech Inc., 8045 Lamon Avenue, Suite 400, Skokie, IL USA

**Keywords:** Carbonic anhydrase, Clostridium autoethanogenum, Gas fermentation, Carbon dioxide, Carbon monoxide, Enzyme characterisation

## Abstract

Carbonic anhydrase catalyses the interconversion of carbon dioxide and water to bicarbonate and protons. It was unknown if the industrial-relevant acetogen *Clostridium autoethanogenum* possesses these enzymes. We identified two putative carbonic anhydrase genes in its genome, one of the β class and one of the γ class. Carbonic anhydrase activity was found for the purified β class enzyme, but not the γ class candidate. Functional complementation of an *Escherichia coli* carbonic anhydrase knock-out mutant showed that the β class carbonic anhydrase could complement this activity, but not the γ class candidate gene. Phylogenetic analysis showed that the β class carbonic anhydrase of *Clostridium autoethanogenum* represents a novel sub-class of β class carbonic anhydrases that form the F-clade. The members of this clade have the shortest primary structure of any known carbonic anhydrase.

**Electronic supplementary material:**

The online version of this article (10.1007/s00253-019-10015-w) contains supplementary material, which is available to authorized users.

## Introduction

*Clostridium autoethanogenum* fixes carbon dioxide through the Wood–Ljungdahl pathway (WLP) and produces acetate, ethanol and 2,3-butanediol natively (Abrini et al. 1994; Köpke et al. 2011). As such, use of *C. autoethanogenum* provides an attractive way to mitigate the effects of global CO_2_ release. Besides capture in the WLP, CO_2_ is also fixed at other metabolic steps, and it was shown, for instance, that elevated CO_2_ partial pressures benefit the production of 2,3-butanediol (Simpson et al. 2014). In fact, many reactions in microbial metabolism exist where CO_2_ or bicarbonate are substrates or products (Smith and Ferry 2000). It was proposed that without a mechanism for the rapid interconversion of carbon dioxide and bicarbonate, the turnover rates of common carboxylation reactions that consume bicarbonate would not be feasible in *Escherichia coli* (Merlin et al. 2003). The interconversion of CO_2_ and bicarbonate (Eq. (1)) is catalysed by carbonic anhydrase (CA), an enzyme that is essential for most forms of life. To optimise product formation and carbon fixation, knowledge about CA activity is important (Hawkins et al. 2013; Lian et al. 2016).

1 $$ {\mathsf{H}}_{\mathsf{2}}\mathsf{O}+{\mathsf{CO}}_{\mathsf{2}}\leftrightarrow {{\mathsf{H}\mathsf{CO}}_{\mathsf{3}}}^{-}+{\mathsf{H}}^{+} $$

There are three major classes of carbonic anhydrase, which are as follows: α-CA, β-CA and γ-CA. The α-class protein is active in monomeric state, while the β-CAs form dimers, which in turn can stack into tetramers or octamers. The γ-CAs form homotrimeric structures (Lindskog 1997; Ferry 2010; Rowlett 2010). In addition, δ-CA, ζ-CA and η-CA classes were proposed for non-canonical CA enzymes present in diatoms and *Plasmodium* sp. (Del Prete et al. 2014). An initially identified ε-CA class of carboxydosome-specific CAs was later reclassified as a subgroup of β-CA after it was recognised that these enzymes were structurally similar, despite little sequence similarity (Sawaya et al. 2006). This specific subclass will be referred to as the E-clade of β-CA in the remainder of this article.

Carbonic anhydrases (EC 4.2.1.1) are metalloenzymes. All known CAs function with a Zn^2+^ metal ion cofactor in their active site, but some are functional with other metal ions as well. In γ-CAs of anaerobic microorganisms, Fe^2+^ or Co^2+^ can functionally replace Zn^2+^, while the ζ-class CA is also functional with Cd^2+^ (Kumar and Ferry 2014; Supuran 2016). The metal ions are coordinated in the active site by three histidines (for α-, γ-, δ- and η-class CAs) or one histidine and two cysteines (for β- and ζ-class CAs). These amino acids are arranged in a distinct motif for each CA class (Hewett-Emmett and Tashian 1996; Ferry 2010; Rowlett 2010; Capasso and Supuran 2015). The diverse class of microbial β-CA is divided in five clades, A–D (Smith and Ferry 2000) plus the former ε-CA class that we will recognise here as the E clade. All β-CA sport the common metal-coordinating active-site motif CxDxR-G-HxxC (Lindskog 1997; Ferry 2010; Rowlett 2010).

The physiological role of CA enzymes is diverse, but not always clear. In animals, they are essential for rapid gas exchange and other functions, such as pH homeostasis. Since specific CAs act in specific tissues with specific inhibition patterns, CAs are common drug targets (Supuran and Scozzafava 2007). In plants and algae, CAs have a function in carbon dioxide diffusion facilitation and carbon concentration mechanisms (Moroney et al. 2001; Moroney et al. 2011). In prokaryotes, CAs are almost ubiquitous. Proposed functions of CAs in prokaryotes are to act in carbon concentration (Cannon et al. 2010), carbon dioxide transport (Gai et al. 2014), facilitation of carbon dioxide or bicarbonate-consuming or -producing reactions (Smith and Ferry 2000; Merlin et al. 2003; Supuran and Ferry 2013), pH homeostasis (Sachs et al. 2005) and acetate transport facilitation (Braus-Stromeyer et al. 1997). Most bacteria can grow under low carbon dioxide partial pressures. For this, a CA gene seems to be essential since species that need high carbon dioxide partial pressures (capnophiles) often have no detectable CA activity and some have lost CA genes (Ueda et al. 2008; Ueda et al. 2012). For the capnophile *Campylobacter jejuni*, it was shown that it contains a CA that is only active at high pH, but not under normal physiological pH (Al-Haideri et al. 2016). Also, CA deletion mutants often can only grow under high carbon dioxide partial pressures (Kusian et al. 2002; Merlin et al. 2003; Kumar et al. 2013), making them functional capnophiles. In *E. coli*, a specific CA, CynT, is part of cyanate metabolism operon that is tightly controlled and induced by cyanate or azide (Guilloton et al. 1993). When the constitutively expressed CA *can* gene of *E. coli* was disrupted, the mutant strain (*E. coli* EDCM636) was unable to grow under atmospheric carbon dioxide pressure. Addition of azide restored its normal growth (Merlin et al. 2003). This strain is useful for studies to test if putative CA genes can complement CA activity.

Production of 3-hydroxypropionate by the expression of bicarbonate-dependent reactions in *Pyrococcus furiosus* that lacks a functional CA benefited greatly from the expression of functional CA genes (Lian et al. 2016) underpinning the importance of CAs in a biotechnological context.

Previously, a diverse set of acetogens, i.e., bacteria using the WLP, screened for CA activity showed a range of CA activities (Braus-Stromeyer et al. 1997). One model of acetogen, *Acetobacterium woodii*, had high CA activity, but the other model of acetogen, *Moorella thermoacetica*, showed little to no activity. No close relatives of *C. autoethanogenum* were included in this CA screening.

Here, we identified two putative CA genes in the genome of *C. autoethanogenum*. One of these encoded a dimeric enzyme that was indeed active as CA. The gene formed a new F clade in the β-class of CA. This new clade represents CAs with the shortest primary structure known.

## Materials and methods

### Bioinformatics

To search the online protein databases, we used NCBI blastp, PSI-Blast, Delta-Blast, PHI-Blast and tblastn algorithms (Sayers et al. 2012). For alignments, we used the MUSCLE algorithm (Edgar 2004) in both Ugene (Okonechnikov et al. 2012) and Mega7.0 (Kumar et al. 2016). Consensus logos were made using JalView (Waterhouse et al. 2009). Phylogenetic analysis was performed in mrBayes (Ronquist et al. 2012) and Mega7.0, and figures were made in Figtree (Rambaut 2014). To find the optimal evolutionary model, we used Prottest (Darriba et al. 2011) and Mega7.0. Structural protein modelling was done by Phyre2 (Kelley et al. 2015). Protparam of the ExPASy server (Gasteiger et al. 2005) was used to calculate predicted parameters of proteins.

### Strains and growth conditions

*C. autoethanogenum* JA1-1 (DSM 10061) was obtained from the Deutsche Sammlung von Mikroorganismen und Zellkulturen GmbH (Germany). *E. coli* EDCM636 was from the Coli Genetic Stock Centre (CGSC, New Haven, CT, USA). *C. autoethanogenum* was grown in YTF medium (yeast extract 10 g l^−1^, tryptone 16 g l^−1^, fructose 10 g l^−1^, NaCl 0.2 g l^−1^) with trace elements (H_3_BO_3_ 100 μg l^−1^, MnCl_2_.4H_2_O 230 μg l^−1^, FeCl_2_.4H_2_O 780 μg l^−1^, CoCl_2_.6H_2_O 103 μg l^−1^, NiCl_2_.6H_2_O 602 μg l^−1^, ZnCl_2_ 78 μg l^−1^, CuSO_4_.5H_2_O 50 μg l^−1^, AlK (SO_4_)_2_.12H_2_O 50 μg l^−1^, Na_2_SeO_3_ 58 μg l^−1^, Na_2_WO_4_ 53 μg l^−1^, Na_2_MbO_4_.2H_2_O 52 μg l^−1^) and vitamins (p-aminobenzoate 57 μg l^−1^, riboflavin 52 μg l^−1^, thiamine 100 μg l^−1^, nicotinate 103 μg l^−1^, pyridoxine 255 μg l^−1^, calcium pantothenate 52 μg l^−1^, cyanocobalamin 39 μg l^−1^, biotin 11 μg l^−1^, folate 24 μg l^−1^, thioctic acid 25 μg l^−1^, at pH 5.8. All cultures of *C. autoethanogenum* were grown at 37 °C in a MG1000 anaerobic workstation with TG airlock (Don Whitley, UK).

All *E. coli* strains were grown on LB medium (tryptone 10 g l^−1^, yeast extract 5 g l^−1^, NaCl 10 g l^−1^). To induce the Cyn operon and thus enable growth of *E. coli* EDCM636, 0.1 mM sodium azide (Az) was added to the medium. Erythromycin (Em) was used at a concentration of 500 μg ml^−1^ and ampicillin at 100 μg ml^−1^ when present.

### Plasmid construction

Enzymes used for subcloning and PCR were purchased from New England Biolabs (NEB, MA, USA). All other chemicals were purchased from Sigma-Aldrich except for Bugbuster (Merck Millipore, Germany) and Strep-Tactin sepharose (IBA, Germany).

Plasmids for heterologous overexpression of CA genes were constructed as follows: Genes were amplified by polymerase chain reaction (PCR) using genomic DNA of *C. autoethanogenum* as template. The genomic DNA was isolated using the Genelute (Sigma) genomic DNA isolation kit. PCR was performed with primers (Table 1) FbCA and RbCA to obtain an untagged version of *caut-bCA* and primers FbCA and RbCAstrep to obtain a C-terminal strep-tagged version of the same gene. Similarly, primers FgCA and RgCA were used to obtain an untagged *caut-gCA* and the primers FgCA and RgCAStrep to obtain a C-terminal strep-tagged version of the *gCA* gene. These PCR fragments were subcloned into pMTL82252 (Heap et al. 2009) using *Nde*I, *Eco*RI and T4-ligase. To construct His-tagged versions of Caut-bCA and Caut-gCA, we performed PCR with primers ΒCAECPF and ΒCAECPR or FPCA1 and RPCA1, respectively. The resulting fragments were cloned into a pET16b (Novagen) plasmid using *Nde*I and *Bam*HI and T4-ligase. *E. coli* DH5α chemically competent cells (NEB) were transformed with these plasmids. Plasmids were isolated using Monarch plasmid isolation kit (NEB) and Sanger sequenced by Eurofins using the FpMTL8xx5x primer for the pMTL82252 plasmids and the pET16b_F primer for the pET16b plasmids.

**Table 1:** Primers used in this study

Primer name	Primer sequence
FgCAOE	TATACATATGATAAGAAAATTTGAACATTAC
RgCAOE	TATAGAATTCTAATATTCACTATAATTTTTAGC
FgCAOEStrepN	TATACATATGTGGTCACATCCTCAATTTGAAAAAATGATAAGAAAATTTGAACATTAC
RgCAOEStrepC	TATAGAATTCTTATTTTTCAAATTGAGGATGTGACCAATATTCACTATAATTTTTAGC
RgCAOEHisC	TATAGAATTCATGATGATGATGATGATGATGATGATTATATTCACTATAATTTTTAGC
FbCAOE	TATACATATGTTGAACAGTGATTTTGCTGTATTGTTAAATTGTATG
RbCAOE	TATAGAATTCTAAAGTTTTTCCACTTCGAAATTCTCATTTATC
FbCAOEStrepN	TATACATATGTGGTCACATCCTCAATTTGAAAAATTGAACAGTGATTTTGCTGTATTGTTAAATTGTATG
RbCAOEStrepC	TATAGAATTCTTATTTTTCAAATTGAGGATGTGACCAAAGTTTTTCCACTTCGAAATTCTCATTTATC
FpMTL8xx5x	GAAGTACATCACCGACGAGC
FPgCA	GTTGTCCATATGATAAGAAAATTTGAACAACATTACATACCAG
RPgCA	GTTGTTGGATCCCTAATATTCACTATAATTTTTAGCCC
ΒCAECPF	GTTGTCCATATGTTGAACAGTGATTTTGCTGTATTG
ΒCAECPR	GTTGTTGGATCCCTAAAGTTTTTCCACTTCG
FPCA1	GTTGTCCATATGATAAGAAAATTTGAACAACATTACATACCAG
RPCA1	GTTGTTGGATCCCTAATATTCACTATAATTTTTAGCCC
pET16b_F	GATCCCGCGAAATTAATACGA

*E. coli* BL21(DE3) pLysS was transformed with pET16b-bCA and pET16b-gCA. *E. coli* EDCM636 was transformed with pMTL82252-bCA, pMTL82252-bCAstrepC and pMTL82252-gCA. The transformed cells were plated on LB agar with Em and on LB agar with Em and Az. Colonies were restreaked on LB agar, LB agar with Em, LB agar with Em and Az and LB agar with Az, to study the ability of the *caut-bCA* and *caut-gCA* genes to complement the ΔCA mutation of *E. coli* EDCM636s.

### Protein purification

To produce and purify the His-tagged putative CA enzymes from the BL21(DE3) cells, 5 ml LB was inoculated from a − 80 °C stock and grown overnight at 225 rpm, 37 °C. This O/N culture was used to inoculate 5 × 100 ml LB in 500 ml Erlenmeyer flasks to a start OD_600_ of approximately 0.05 and incubated in a shaking incubator at 225 rpm, 37 °C. At OD_600_, 0.3–0.7 cells were induced with IPTG (Isopropyl β-D-1-thiogalactopyranol), a 0.5 mM final concentration, and incubated at 30 °C for 3–5 h. Cells were harvested by centrifugation, and cells were lysed with a QS1 probesonicator (Nanolabs, MA, USA) or with BugBuster® Plus Lysonase™. An additional centrifuge step was used to create cell-free extract. The protein was purified using a 5 ml HisTrap® HP collumn (GE Healthcare Life Sciences, Buckinghamshire, UK) and 300 mM imidazole for elution buffer. For the production of the STREP-tagged enzyme, 300 ml of overnight culture (LB, shaking, 37 °C) *E. coli* EDCM636 pMTL82252-bCAstrepC or EDCM636 pMTL82252-gCAstrepC was harvested by centrifugation and lysed using BugBuster Plus Lysonase with Avidin added at one small crystal per 30 ml. The Caut-bCA protein was purified using Strep-Tactin Sepharose using 100 mM Tris pH 8 with 150 mM NaCl as wash buffer. For elution, 0.5 mg ml^−1^ desthiobiotin was added to the wash buffer. Samples of all purification fractions were resolved on a NuPAGE Novex 4–12% Bis–Tris Protein Gels (ThermoFisher, Waltham, MA, USA) to assess purity and yield. Protein concentration was further analysed using a NanoDrop Lite photospectrometer (Thermo-Scientific, Wilmington, DE, USA).

### Analytical ultracentrifugation

For characterisation of the purified Caut-bCA by analytical ultracentrifugation, sedimentation velocity scans were recorded for a 2-fold protein dilution series, starting at 0.7 mg ml^−1^. The analysis was performed at 50,000 rpm, using a Beckman XL-I analytical ultracentrifuge with an An-50Ti rotor, and results were obtained by absorbance measured at 280 nm and interference optical detection systems. The density and viscosity of the buffer were measured using a DMA 5000 M densitometer equipped with a Lovis 200ME viscometer module. The partial specific volume for the protein was calculated using Sednterp from the amino acid sequence. Data were processed using SEDFIT, fitting to the c(s) or non-interacting discrete species (NIDS) model (Schuck 2000).

### CA activity assay

To determine CA activity, we developed an assay using a Tecan M1000-Pro (Männedorf, Switzerland) plate reader with auto injector, based on previous methods (Wilbur and Anderson 1948; Sundaram et al. 1986; Fasseas et al. 2011; Gai et al. 2014). We have validated the assay using bovine CA (Sigma) and *C. jejuni* CanB (Al-Haideri et al. 2016) (kindly supplied by D.J. Kelly of the University of Sheffield). The assay buffer was 50 mM HEPES, 50 mM Na_2_SO_4_, 50 mM MgSO_4_, 0.004% (*w*/*v*) phenol red at several pH values. The substrate for the hydration reaction was carbon dioxide-saturated water, produced by bubbling carbon dioxide through demineralised water at 20 °C for 30 min. This should result in 34 mM CO_2_ (Diamond and Akinfiev 2003); for lower concentrations, the CO_2_-saturated water was diluted with demineralised water that was sparged with N_2_ for 30 min. The substrate of the dehydration reaction was KHCO_3_ at 100 mM. For the hydration reaction, 120 μl of assay buffer was mixed with 10 μl enzyme sample or 10 μl sample buffer in a 96-well plate. After measuring the baseline for 4 s, 120 μl of substrate was injected. For the dehydration reaction, 140 μl and 10 μl enzyme samples or 10 μl sample buffer was used, after 4 s baseline measurement 50 μl substrate was added. Change of absorption was measured at 557 nm for 40 s at a temporal resolution of 200 ms. The average change in absorption s^−1^ of the first ten readings was taken as the initial speed of the reaction. The *Km* and *V*max were calculated using the Michaelis and Menten (1913) curve fitting tool of GraphPad Prism 7.00 (La Jolla, CA, USA).

## Results

To establish if *C. autoethanogenum* harbours CA genes in its genome, we collected 41 CA protein sequences covering the α-, β-, γ- and δ-CA classes of organisms from all domains of life. We assembled consensus sequences of the α-, β- and γ-CA classes and then used amino acid sequences and the assembled consensus sequences to search the genome of *C. autoethanogenum*. One gene that encoded a putative γ-CA (Caut-gCA, CLAU_2699) was found with a PSI-BLAST E-value of 3e-50 and 54% sequence identity to the γ-CA consensus sequence. This gene was annotated as a hexapeptide repeat-containing protein, a feature that exists in γ-CA (Iverson et al. 2000). It had the three important metal-binding histidine residues conserved but lacks glutamine and asparagine residues that were found to be important in the canonical γ-CA (Cam) from *Methanosarcina thermophile* (Ferry 2010)*.* A gene coding for a putative β-CA (Caut-bCA, locus tag CLAU_3021) was found using the β-CA consensus sequence in PSI-BLAST and matched with an *E* value of 1e−80. Despite the low similarity to other β-CAs, this gene encodes a protein that contains the motifs CxDxR and HxxC, which are known to be important for the activity of β-CAs (Smith and Ferry 2000; Smith et al. 2002).

In recently published transcriptomics data, *caut-bCA* is consistently higher expressed than *caut-gCA* (148.177 FPKM vs 71.7564 FPKM) (Marcellin et al. 2016). Downstream of the *caut-gCA* gene, a peptidase M14 carboxypeptidase was present, which has no obvious link to CAs except that both are Zn metalloenzymes. The *caut-bCA* gene was part of a cluster (CLAU_3019-CLAU_3023) of five genes with similar expression pattern (unpublished RNAseq data). A *yopX* gene and a single-stranded DNA-binding protein-encoding gene that are present in this cluster indicate a phage origin. The gene immediately downstream of *Caut-bCA* was annotated as a deoxyuridine 5′-triphosphate nucleotidohydrolase which indicates a potential function in pyrimidine metabolism where HCO_3_^−^ is an important factor.

Protparam results showed that the 124 amino acid residues of Caut-bCA formed a stable protein with a molecular weight of 14.2 kDa and pI of 5.3. The 168 amino acid residues of Caut-gCA were expected to form an unstable protein with a mass of 18.3 kDa and pI of 6.4. Phyre2 structural modelling showed Caut-bCA as a small but not untypical β-CA with an accessible active site.

### Functional complementation of E. coli Can<FLK2>

**Fig. 1:**
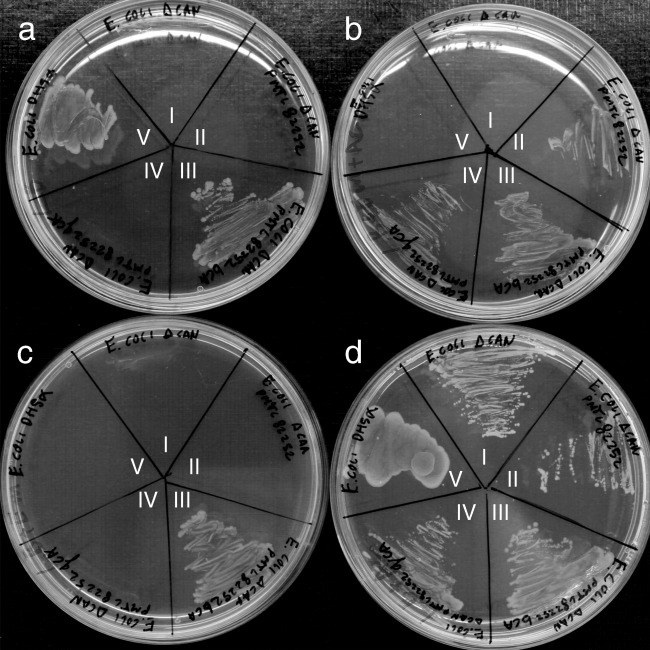
Complementation of *E. coli* EDCM636 with Caut-bCA and Caut-gCA. Strains of *E. coli* EDCM636 transformed with caut-bCA or caut-gCA on plasmid, and control strains were streaked on LB agar medium (**a**), LB with erythromycin and azide (**b**), LB with erythromycin (**c**) and LB with azide (**d**). *E. coli* strains indicated by roman numerals were as follows: (I) EDCM636 (ΔCan), (II) EDCM636 (ΔCan)-pMTL82252, (III) EDCM636 (ΔCan)-pMTL82252-bCA, (IV) EDCM636 (ΔCan)-pMTL82252-gCA and (V) DH5α

The *Can* disruption mutant of *E. coli* EDCM636 (Merlin et al. 2003), which lacks a constitutively expressed CA and is therefore unable to grow under atmospheric carbon dioxide pressure, was used to test if Caut-bCA and Caut-gCA exhibited CA activity. When transformed with plasmids carrying the *caut-bCA* or *caut-gCA* gene, only *E. coli* EDCM636 complemented with *caut-bCA* was able to grow in the absence of azide (Fig. 1). *E. coli* EDCM636 cells transformed with pMTL82252-bCAstrepC also grew without the absence of azide, indicating the C-terminal strep tag did not impair activity of the enzyme. We did find that *E. coli* EDCM636 cells infrequently reverted to the native phenotype and grow in the absence of azide. Therefore, we repeated this experiment three times with fresh batches of competent cells that did not show WT phenotypes.

### Development of a high-throughput CA activity assay method

To establish whether the identified genes encode for active CA enzymes, we developed a high-throughput activity assay that was performed in 96-well format. As far as the authors know, this is the first description of performing CA assays in 96-well format. The performance of the assay was assessed with bovine α-CA with a dose–effect response in the range of 0.003–3 μg ml^−1^ of enzyme. It was further validated by measuring the *K*_M_ of bovine α-CA and the pH-specific activity of the *C. jejuni* CanB, a β-CA (Al-Haideri et al. 2016). The method replicated the specific pH profile of *C. jejuni* CanB, typical of type II β-CAs, and the *K*_M_ was determined as 4.7 ± 2 mM at 20 °C. Al-Haideri et al. found a *K*_M_ of 34 ± 10 mM at 4 °C; however, this temperature could not be replicated in our Tecan M1000-Pro. The *K*_M_ of bovine CA was determined as 17 ± 4 mM while published values vary between 12 and 1.1 mM (Kernohan 1964; Iqbal et al. 2014). We continued to apply this assay in the characterisation of *C. autoethanogenum* CAs.

### Enzyme characterisation

**Fig. 2:**
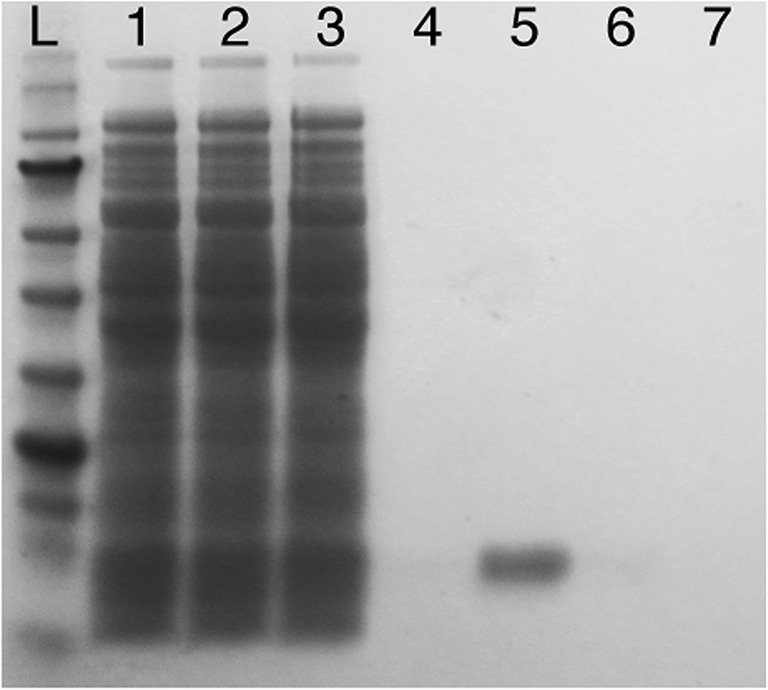
SDS-PAGE analysis of Caut-bCAstrepC protein purification fractions. Lane L, NEB protein ladder P7712; lane 1, lysate; lane 2, column flow through; lane 3, initial wash; lane 4, final wash; lane 5, first eluate; lane 6, second eluate; lane 7, third eluate

**Fig. 3:**
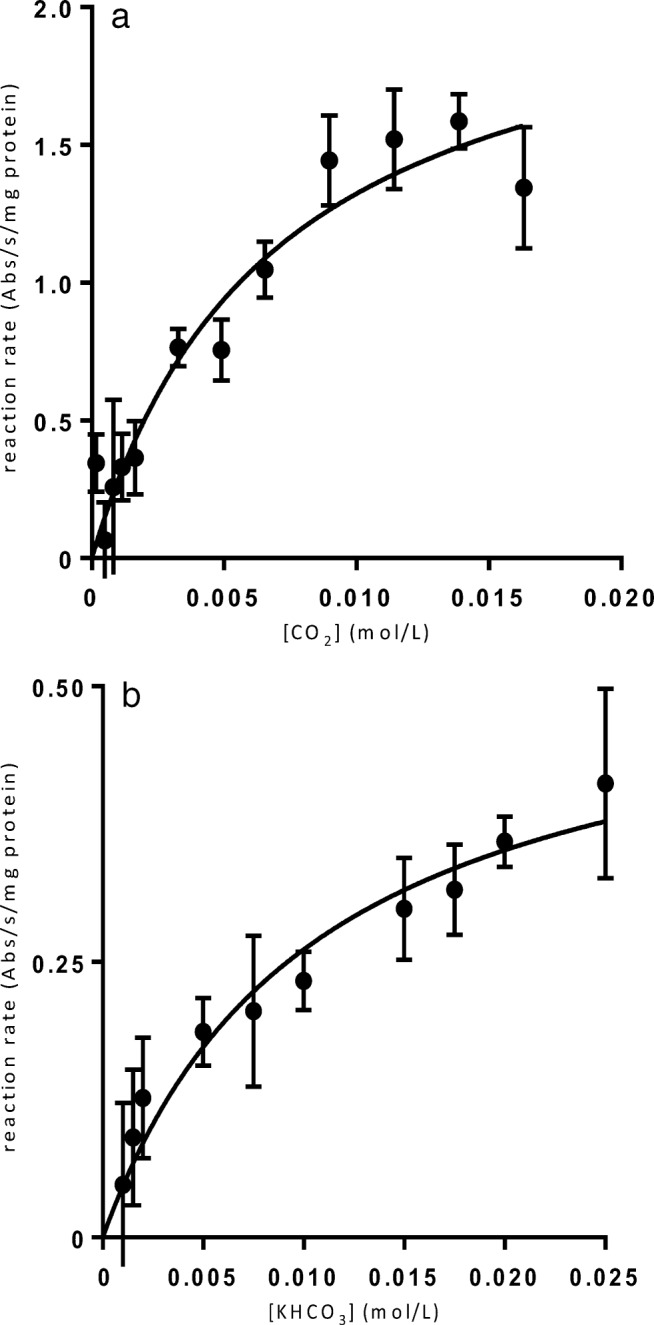
The reaction rates of CO_2_ hydration (left) and KHCO_3_ dehydration (right) are shown. The values are the difference between the uncatalysed and catalysed rates measured by absorption at 557 nm. The enzyme was assayed in a buffer of 50 mM HEPES, 50 mM MgSO_4_, 50 mm Na_2_SO_4_, 0.004% (*w*/*v*) phenol red pH 8.3 with CO_2_ as a substrate, and pH 6 for KHCO_3_^−^ substrate at 20 °C. The data points represent the mean and SD, *N* ≥ 4. The curve is the fit to the Michaelis–Menten equation

To characterise the specific activity of the putative CAs of *C. autoethanogenum*, they were heterologously expressed, purified and assayed for the activity. Purification of the Caut-bCA and Caut-gCA with N-terminal His-tag (Hochuli et al. 1988) on a pET16b plasmid expressed in BL21(DE3) pLysS cells did not reliably yield active enzymes (data not shown). A C-terminal STREPII-tagged (Schmidt and Skerra 2007) Caut-bCA protein, expressed from pMTL82252 in the *E. coli* EDCM636 strain, was reliable purified (Fig. 2) and used for further characterisation of the enzyme. We did not manage to show CA activity for Caut-gCA or Caut-gCAstrepC with any of the tested systems and did not further characterise this enzyme. The developed high-throughput CA assay method was used to measure the kinetic parameters of the hydration reaction of the Caut-bCAstrepC enzyme (Fig. 3). The *K*_M_ for the hydration reaction was measured at 6.8 ± 1.6 mM, and, for the dehydration reaction, it was 10.5 ± 2.5 mM. The measured kinetic parameters of the hydration reaction were comparable with those of the other reported β-CAs (Table 2), and comparative data is mostly lacking for the dehydration reaction. Incubation of the purified enzyme at 95 °C for 10 min caused total loss of activity (data not shown).

**Table 2:** Kinetic parameters of Caut-bCA and previously described beta-carbonic anhydrases

Species	Monomer size (kDa)	Oligomeric state	Clade	Hydration		Dehydration		Reference
				*K*_cat_ (s^−1^)	*K*_cat_/*K*_M_ (s^−1^ M^−1^)	*K*_cat_ (s^−1^)	*K*_cat_/*K*_M_ (s^−1^ M^−1^)	
*Clostridium autoethanogenum*	14.2	Dimer	F	2.1 × 10^5^	3.1 × 10^7^	6.3 × 10^4^	6.0 × 10^6^	This study
*Pisum sativum*	24.2	Octamer	B	4 × 10^5^	1.8 × 10^7^	NA	NA	Kisiel and Graf (1972); Johanson and Forsman (1993)
*Cryptococcus neoformans*	26	Dimer	A	3.9 × 10^5^	4.3 × 10^7^	NA	NA	Innocenti et al. (2008)
*Drosophila melanogaster*	30.0	Dimer	B	*9.5 × 10* ^*5*^	1.1 × 10^8^	NA	NA	Syrjänen et al. (2010)
*Clostridium perfringens* strain 13	21.3	Tetramer	D	1.5 × 10^4^	4.8 × 10^6^	NA	NA	Kumar et al. (2013)
*Methanobacterium thermautotrophicus*	18.9	Tetramer	D	1.7 × 10^4^	5.9 × 10^6^	NA	NA	Smith and Ferry (1999)
*Halothiobacillus neapolitanus*	57.3	Dimer	E	8.9 × 10^5^	2.8 × 10^7^	4.6 × 10^4^	4.9 × 10^6^	Sawaya et al. (2006); Heinhorst et al. (2006)
*Salmonella enterica* (*stCAI*)	24.8	NA	A	7.9 × 10^5^	8.3 × 10^7^	NA	NA	Nishimori et al. (2011)
*Salmonella enterica* (*stCAII*)	26.6	NA	C	8.9 × 10^5^	5.2 × 10^7^	NA	NA	Nishimori et al. (2011)

NA, not available

**Fig. 4:**
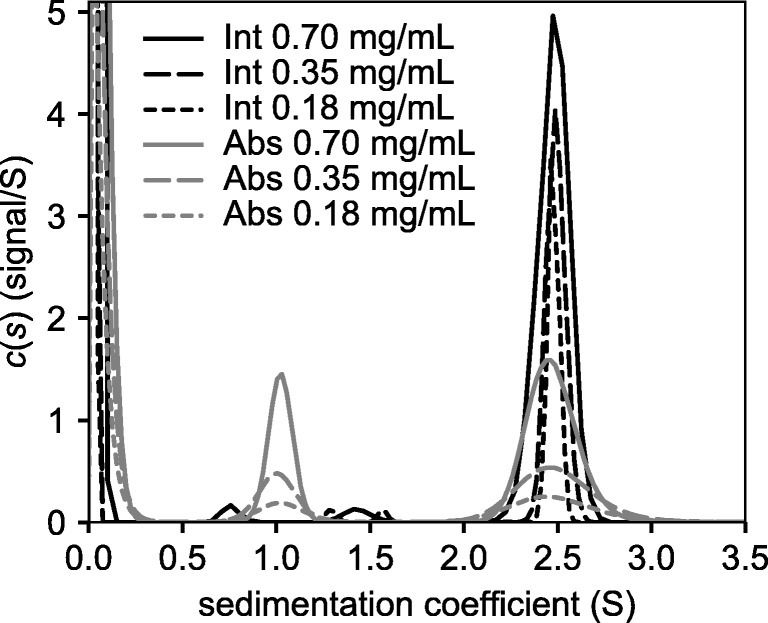
Analytical ultracentrifuge analysis of Caut-bCAstrepC. c(s) distributions for Caut-bCAstrepC. Int, interference data; Abs, absorbance data. (The sharp peak at ~ 0.1S is buffer salt)

Analytical ultracentrifugation of Caut-bCAstrepC (Table 3 and Fig. 4) showed that molecular weight of 30 kDa obtained for the main peak of the derived sedimentation coefficient distribution was consistent with that of a dimer.

**Table 3:** Caut-bCAstrepC estimated molecular weights from c(s) and NIDS analysis by analytical ultracentrifugation

			Major species				
Monomer MW (kDa)	Detection method	Concentration (mg/mL)	Peak 1		Peak 2		
			MW (kDa)	Sed. co (S)	MW (kDa)	Sed. Co (S)	*f*/*f*_0_^a^
C(s)							
		0.70	9.3	1.03	34.6	2.48	1.44
	Absorbance	0.35	8.4	1.01	32.8	2.49	1.37
		0.18	9.3	1.02	35.1	2.48	1.45
15.2		0.70	–	–	31.3	2.48	1.29
	Interference	0.35	13.0	1.45	29.3	2.49	1.23
		0.18	11.9	1.32	30.6	2.48	1.27
NIDS							
		0.70	10.1	1.04	29.3	2.49	N/A
15.2	Absorbance	0.35	9.7	1.02	29.4	2.51	N/A
		0.18	3.6	0.94	29.8	2.49	N/A

^a^Best-fit frictional ratio *f*_0_

### Phylogeny

**Fig. 5:**
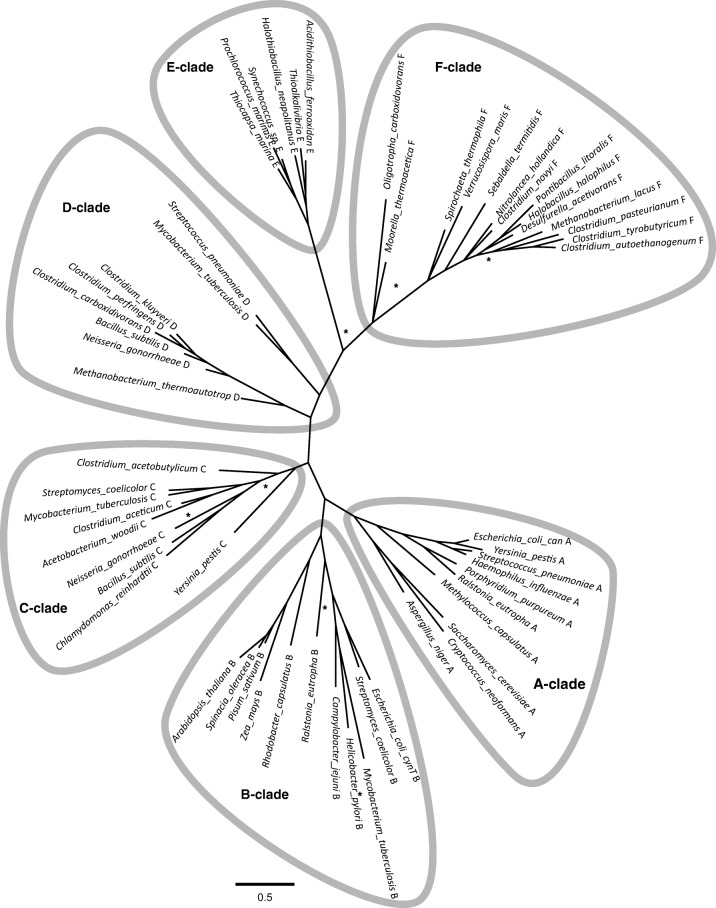
β-CA phylogeny. The phylogeny of β-CAs was reconstructed using MrBayes. All posterior probabilities are above 0.85 except for branches labelled with * which have a probability between 0.55 and 0.7. A similar topology was obtained with more extended sets of sequences, both with maximum Likelihood method and Bayesian analysis (data not shown). The tree was edited for clarity using Figtree and Inkscape

The low identity of the putative Caut-bCA with known β-CAs triggered further phylogenetic analysis. A Bayesian phylogenetic tree of 60 β-CA sequences was constructed (Fig. 5) which present the overall topology of identified clades, consistent with that found with other methods (maximum likelihood) and larger sets of β-CA sequences (data not shown). The analysis included sequences of the majority of previously described β-CAs as well as β-CAs from the major taxonomic groups of life. We identified six major clades within the β-CAs (Fig. 5). The A, B, C and D clades are as described previously (Smith and Ferry 2000), and the E clade was formed by the former ε-CAs. The Caut-bCA-like proteins formed a distinct F clade, of not previously studied β-CAs.

An alignment of 160 β-CA protein sequences was summarised as consensus logos for all six identified subclades (Online Resource 1). Only two motifs interspaced by 48–77 amino acids were fully conserved in all selected β-CAs. These were the HxxC motifs, which bind the active site metal ion and the CxDxR motif that completes the active site (Rowlett 2010). Additional motifs were identified for subgroups of the clades. Enzymes in the A, B and C clades all contained a QxP motif of six amino acids N-terminal of the CxDxR motif, while D and E clades lack this motif. In most of the novel F clade, the QxP motif was found on the C-terminal side of the CxDxR motif. The A, B and C clades further contained a conserved G [D/E] xFxR motif in sequence that was flanked by the CxDxR and HxxC motifs. In the E clade, only GxxF was conserved, and in the D clade, G [D/E] was conserved. The distance between CxDxR and HxxC was larger than average in the E clade with approximately 75 aa, and to a lesser extent in the F clade with average distance of 64 aa, where A, B, C and D clades had an average distance of 55 aa.

Both phylogenetic and alignment analysis showed that the F clade was further split in two sub-clades, one defined by the putative β-CA of *M. thermoacetica* (Mtherm-bCA-like) and the other by Caut-bCA. F clade CAs were mostly of shorter sequence than the previously described CAs of the other clades. The Mtherm-bCA-like subclade harbours six proteins of considerable length (172–183 aa). The Caut-bCA-like clade consists solely of relatively short CAs with a length of between 124 and 142 residues.

## Discussion

Two putative CA genes, Caut-bCA and Caut-gCA, were identified in the genome of *C. autoethanogenum* as potential members of the β- and γ-classes of CA. However, a low sequence similarity of Caut-bCA with other members of the β-CA and a reported lack of CA activity for several γ-CA homologs (Martin et al. 2009; Al-Haideri et al. 2016; Kaur et al. 2010; Ferry 2010), did not allow to ascribe CA function to either of these genes based on gene sequence alone. Neither transcription profile nor genomic contexts of the identified genes revealed a specific function for these genes. To assess CA activity for the proteins encoded by these genes, these were heterologously expressed in a *Can* disruption mutant of *E. coli* (strain EDCM636). This showed that Caut-bCA could complement the lack of CA activity in this strain while Caut-gCA did not. This confirmed that Caut-bCA is a carbonic anhydrase while Caut-gCA is not a functional CA in this context. The results of activity assays on purified Caut-gCA and Caut-bCA proteins were consistent with this complementation study as no tested gCA construct yielded any activity while the pMTLCaut-bCAstrepC constructs yielded active purified enzymes. We suspect that the bulky metal binding 10× His residue close to the metal ion-binding active site interfered with the activity of Caut-bCA N-terminal His-tag (Hochuli et al. 1988) on a pET16b plasmid expressed in BL21(DE3) pLysS cells and therefor did not reliably yield active enzyme. Since *E. coli* EDCM636 does not grow without Caut-bCA complementation, Caut-bCA must be produced as an active enzyme. We suspect that this further enhanced the reproducibility of this system.

The measured kinetic parameters were similar to other reported β-CAs. The *K*_M_ of 6.8 mM was slightly higher than that for the β-CAs of *Clostridium perfringens* and *Methanothermobacter thermautotrophicus* but lower than those of *Salmonella enterica* or *Helicobacter pylori.* The particular low *K*_M_ of the *C. perfringens* CA is interpreted as an indication for a function in retaining intracellular levels for anaplerotic CO_2_ fixation reactions (Kumar et al. 2013).

The molecular weight of ~ 30 kDa determined by analytical ultra-centrifuge shows that Caut-bCA is a small dimeric protein. A *Rhodospirilum rubrum* CA was purified with a similar size (28 kDa) and oligomeric state (dimer); however, no matching gene or protein sequence was identified, and later analysis of this species of β-CA showed larger sizes for its β-CA monomers (Gill et al. 1984; Smith et al. 1999). This makes Caut-bCA the smallest confirmed β-CA. The measured molecular weight of the dimer is close to some of the monomers of β-CAs of other species, illustrating the compact nature of Caut-bCA (Table 2).

Because of the low identity of the putative Caut-bCA with known β-CAs, further phylogenetic analysis was performed. We identified multiple orthologs of Caut-bCA in both bacteria and archaea annotated as hypothetical genes. It was striking to find these specific Caut-bCA-like CA sequences relatively often in the deposited genomes of uncultivated bacteria and archaea or recent isolates of candidate species. However, we limited our further phylogenetic analysis to previously cultured species. The phylogenetic analysis revealed that the group of Caut-bCA-like CAs forms a distinct F clade of not previously studied β-CAs that are somewhat distantly related to the other β-CAs of the A, B, C and D subclasses. The further split of the F clade in two subclades (Mtherm-bCA-like and Caut-bCA-like) might be of consequence since *M. thermoacetica* showed little CA activity in previous studies (Braus-Stromeyer et al. 1997). Therefore, some caution is in place to assume functionality on the Mtherm-bCA-like proteins without further study. Based on the similarity of the Caut-bCA-like CAs to Caut-bCA, we assume that these are active CAs. Knowledge on CA in *C. autoethanogenum* could be used to improve CO_2_ utilization or to modulate product spectrum in vivo and for CO_2_ capture and storage in vitro (Alvizo et al. 2014; Warden et al. 2015; Lian et al. 2016).

## Electronic supplementary material

ESM 1 (PDF 1271 kb)
