# Supplementary material for: The carbonic anhydrase of Clostridium autoethanogenum represents a new subclass of β-carbonic anhydrases
Source: Appl Microbiol Biotechnol. 2019 Jul 25;103(17):7275–86. doi: 10.1007/s00253-019-10015-w (PMC6690855; doi:10.1007/s00253-019-10015-w)

Online Resource 1

Applied Microbiology and Biotechnology

The carbonic anhydrase of Clostridium autoethanogenum represents a new subclass of β-carbonic anhydrases.

Bart Pander1, Gemma Harris2, David J. Scott2,3,4, Klaus Winzer1, Michael Köpke5, Sean D. Simpson5, Nigel P. Minton1, Anne M. Henstra1*

1 Clostridia Research Group, BBSRC/EPSRC Synthetic Biology Research Centre, School of Life Sciences, University of Nottingham, Nottingham, NG7 2RD, UK
2 Research Complex at Harwell, Rutherford Appleton Laboratory, Harwell Science and Innovation Campus, Didcot, OX11 0FA, UK
3 ISIS Spallation Neutron and Muon Source, Rutherford Appleton Laboratory, Harwell Science and Innovation Campus, Didcot, OX11 0QX, UK
4 School of Biosciences, University of Nottingham, Sutton Bonington Campus, Sutton Bonington, LE12 5RD, UK.
5 LanzaTech Inc., 8045 Lamon Avenue, Suite 400, Skokie, IL, USA
*anne.henstra@nottingham.ac.uk

Caption

Consensus logos for the different clades (A, B, C, D, E, and F) of β-CA based on an alignment of 160 β-CA sequences. Fm indicates the *M. thermoacetica* subclade and Fc the *C. autoethanogenum* subclade. The complete sequence of Caut-bCA is depicted, other sequences shown were aligned to this sequence. Both ends of sequences were clipped with numbers on the left indicating the number of preceding residues and the numbers on the right the total length of the sequence.

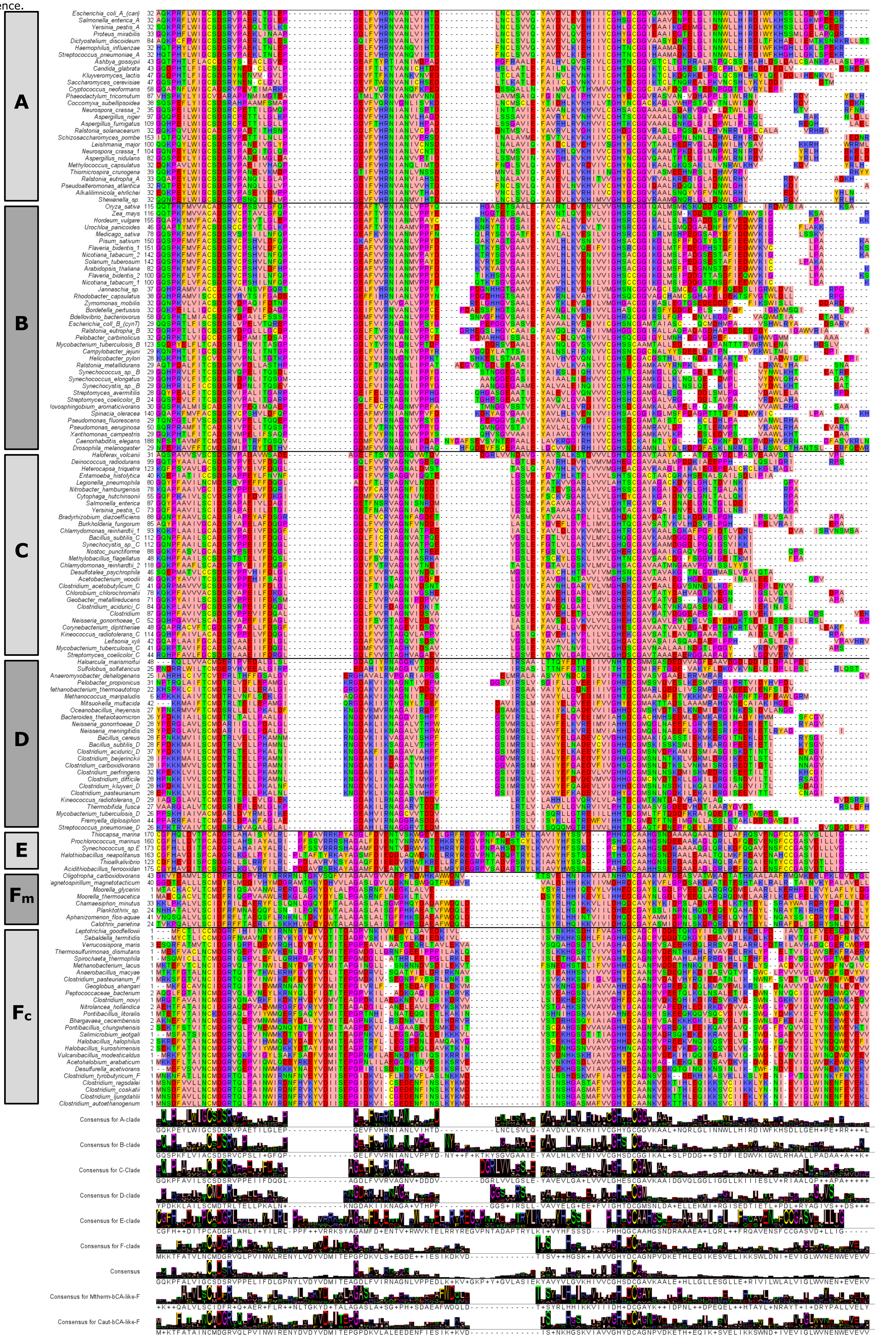

Supplement: Supplementary file 1 — (PDF 1271 kb) [file 253_2019_10015_MOESM1_ESM.pdf]
